# Computational prediction of the human-microbial oral interactome

**DOI:** 10.1186/1752-0509-8-24

**Published:** 2014-02-27

**Authors:** Edgar D Coelho, Joel P Arrais, Sérgio Matos, Carlos Pereira, Nuno Rosa, Maria José Correia, Marlene Barros, José Luís Oliveira

**Affiliations:** 1 Department of Electronics, Telecommunications and Informatics (DETI), Institute of Electronics and Telematics Engineering of Aveiro (IEETA), University of Aveiro, Aveiro, Portugal; 2 Department of Informatics Engineering (DEI), University of Coimbra, Coimbra, Portugal; 3 Centre for Informatics and Systems of the University at Coimbra (CISUC), University of Coimbra, Coimbra, Portugal; 4 Department of Informatics Engineering and Systems, Polytechnic Institute of Coimbra, Engineering Institute of Coimbra (IPC-ISEC), Coimbra, Portugal; 5 Department of Health Sciences, Institute of Health Sciences, The Catholic University of Portugal, Viseu, Portugal; 6 Centre for Neurosciences and Cell Biology, University of Coimbra, Coimbra, Portugal

**Keywords:** Protein-protein interactions, Oral interactome, Bayesian classification

## Abstract

**Background:**

The oral cavity is a complex ecosystem where human chemical compounds coexist with a particular microbiota. However, shifts in the normal composition of this microbiota may result in the onset of oral ailments, such as periodontitis and dental caries. In addition, it is known that the microbial colonization of the oral cavity is mediated by protein-protein interactions (PPIs) between the host and microorganisms. Nevertheless, this kind of PPIs is still largely undisclosed. To elucidate these interactions, we have created a computational prediction method that allows us to obtain a first model of the Human-Microbial oral interactome.

**Results:**

We collected high-quality experimental PPIs from five major human databases. The obtained PPIs were used to create our positive dataset and, indirectly, our negative dataset. The positive and negative datasets were merged and used for training and validation of a naïve Bayes classifier. For the final prediction model, we used an ensemble methodology combining five distinct PPI prediction techniques, namely: literature mining, primary protein sequences, orthologous profiles, biological process similarity, and domain interactions. Performance evaluation of our method revealed an area under the ROC-curve (AUC) value greater than 0.926, supporting our primary hypothesis, as no single set of features reached an AUC greater than 0.877. After subjecting our dataset to the prediction model, the classified result was filtered for very high confidence PPIs (probability ≥ 1-10^−7^), leading to a set of 46,579 PPIs to be further explored.

**Conclusions:**

We believe this dataset holds not only important pathways involved in the onset of infectious oral diseases, but also potential drug-targets and biomarkers. The dataset used for training and validation, the predictions obtained and the network final network are available at http://bioinformatics.ua.pt/software/oralint.

## Background

The majority of gene products that crowd a living cell interact, at least transiently, with other protein molecules. Virtually all cellular events, such as signal transduction, intracellular transport, DNA replication, transcription, translation, splicing, secretion, cell cycle control and intermediary metabolism, are mediated by protein-protein interactions (PPIs) [1]. The same applies to host-pathogen systems, where PPIs are essential in the establishment of infection [2]. The binding domains of interacting proteins reveal high structural and physical-chemical affinity with an associated degree of conservation. This is further evidenced by the fact that close protein homologs frequently interact in the same way [3-7]. With this in mind, we can expect understanding of the human interactome to provide insight into physiopathological mechanisms [8].

Numerous experimental techniques have been explored to attain the human interactome: two-hybrid screening [9,10], affinity purification mass spectrometry [11], DNA microarrays [12], protein microarrays [13-15], synthetic lethality [16], phage display [17], X-ray crystallography and nuclear magnetic resonance spectroscopy [18], fluorescence resonance energy transfer [19], surface plasmon resonance [20], atomic force microscopy [21], and electron microscopy [22]. These methods have major drawbacks that render them non-applicable in large-scale PPI prediction, namely the amount of time, associated cost and minimal protein interaction network coverage per run. Additionally, high-throughput approaches are also often associated with low-specificity and large numbers of both false negatives and false positives [23]. Moreover, these techniques were developed to detect intra-species PPIs, which renders them sub-optimal in inter-species PPI identification. Still, experimental methods remain the only viable methodology to validate PPIs.

As an alternative to experimental methods, a wide range of computational approaches for the prediction of intra-species PPIs have been proposed. Computational methods can be categorized according to the types of information they analyze. One common approach consists of using text mining to extract known PPIs from the biomedical literature [24]. Additionally, there are methods based on genomic data (gene neighborhood [25-28], gene fusion [29,30], phylogenetic profiles [31-33], codon usage similarity [34]), protein structure (homology-based method [35], threading-based method [36]), domain information (single domain pairs [37-41], multi-domain pairs [42,43]), protein sequence [44-56], and Gene Ontology (GO) [57] annotation semantic similarity ([58-61]). In contrast, computational efforts to predict inter-species PPIs have been very limited. Dyer *et al.*[2] combined domain information with a maximum likelihood estimator algorithm [37], while Davis *et al.*[62] adapted an approach following the threading-based method [36]. To provide a better prediction, Tastan *et al.*[63] applied a method combining multiple data sources, and used a random forest classifier to predict interactions between HIV-1 virus and human proteins. Despite these advances, the interactomes of several species are still far from complete. Nonetheless, the results of some of these studies provide great working knowledge of the characteristics of protein and gene interaction networks. For instance, the topological characteristics of protein interaction networks (PINs) have been proven to reflect the functionality of the interacting genes. This was demonstrated in yeast, where essential genes were more likely to be well connected and globally centered in the PIN [64,65].

Here we present a computational model to predict inter-species PPIs within the human oral cavity, an environment particularly prone to bacterial colonization. This is mostly due to the fact that human, microbial and environmental factors interact in a dynamic equilibrium within the human oral cavity [66]. Determination of the salivary interactome will clarify the role of saliva in oral biology and enable the identification of disease biomarkers. The presence of blood exudate proteins and exfoliated epithelial cells in saliva suggest it may be an alternative to blood as a diagnostic fluid in many instances. Additionally, if we consider the systemic nature of saliva, the ease and low cost associated with its handling, and the minimal risk linked to its collection for both medical staff and the patient, the reason for studying the oral cavity becomes clear [67].

As a result of this work, analysis of the resulting PPI network revealed some interesting features. Some of the PPIs involving the *Rothia mucilaginosa* microorganism are very specific and relevant. Moreover, our method not only predicted new PPIs between periodontal pathogens and the host, but also PPIs between different periodontal pathogens, suggesting a synergistic course of action.

## Results

We conducted a series of pre-test analyses to assess the performance of our model. Then, we proceeded to test our approach on high-quality experimental protein-protein interaction (PPI) data collected from five databases. The selected databases exclusively contain manually curated PPI data.

### Computational model for predicting the human-microbial interactome

**Figure 1:**
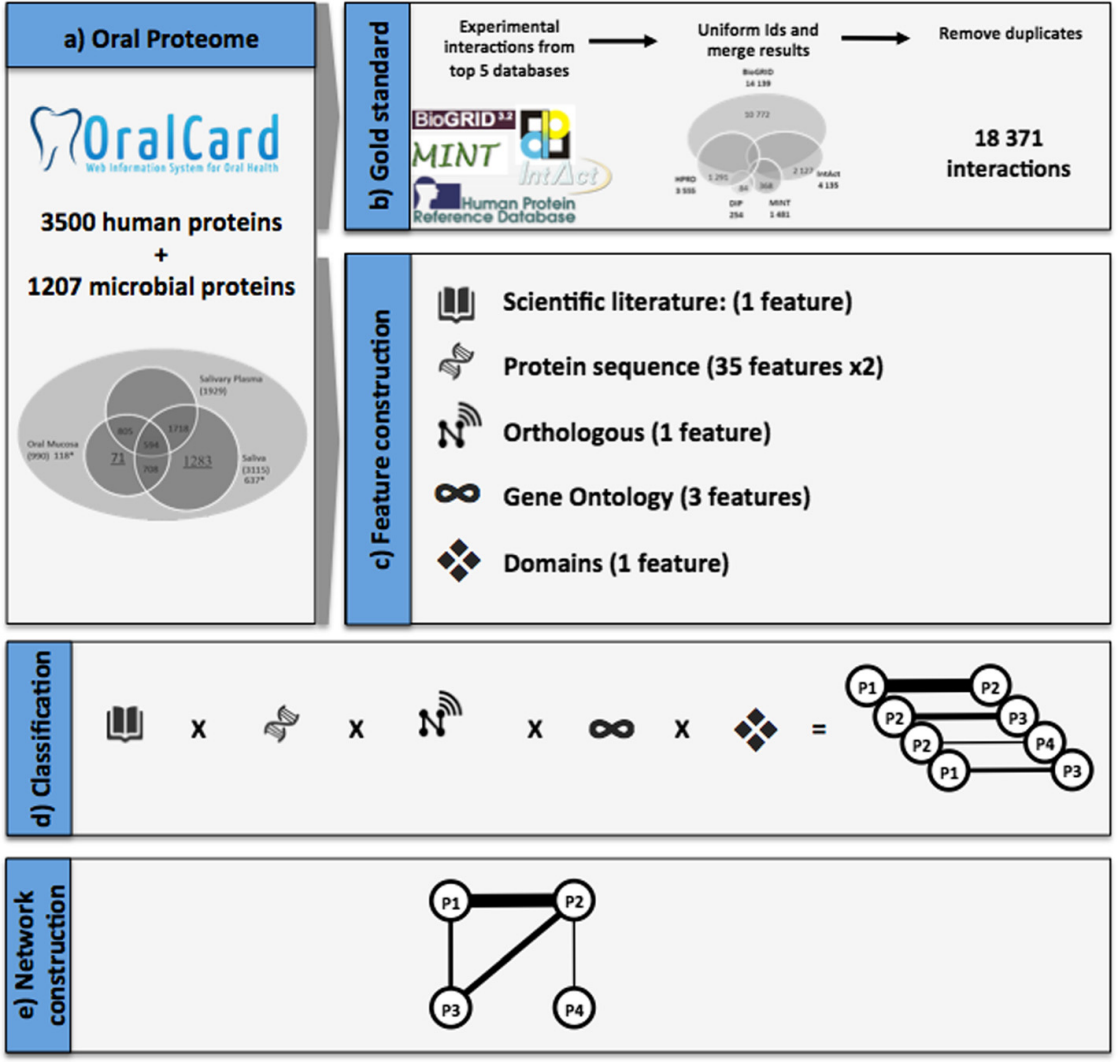
**Workflow applied on the construction of the Human-microbial oral interactome”.** It also contains footnote information: “**a)** the proteins identified on the oral proteome are obtained from the Oralcard database; **b)** the gold standard used for training and validation is obtained by combining the five most relevant curated protein interaction databases; **c)** for each protein interacting pair five clusters of features are constructed; **d)** the previously trained classifier is applied to each pair of interaction; and **e)** finally the interactome network is obtained by combining the individual pairs of proteins.

Figure 1 summarizes the procedure used to achieve the model of the human-microbial interactome. The starting point of this work is a set of 4,707 proteins identified by proteomic studies as being present in the oral cavity and available on the OralCard database [66,67].

Since there is no well-established gold standard for PPIs, we collected data from five databases containing high-quality experimentally determined interactions as described further on in Methods. Extracted PPIs from the five databases were merged, creating our gold standard of positive interactions. The gold standard of negative interactions was obtained by randomly pairing the protein list on the premise that all protein pairs produced must differ from those on the positive dataset. A total of 18,371 positive and a similar number of negative pairs were obtained.

Simultaneously, for each possible pair of proteins, we constructed five clusters of features based on: (1) literature; (2) primary protein sequence information; (3) orthologous profiles; (4) biological process similarity, and; (5) enriched conserved domain pairs. This was performed by accessing public databases, extracting, and then processing the collected data.

The gold standard dataset was used to train a Naïve Bayes classifier and to perform further validations on the final model. The classifier was then applied to the set of all possible pairs of protein interactions. Finally, by aggregating all individual pairs of predicted interactions, the final network was obtained.

### Evaluating the reconstruction of the human interactome

In this section, we evaluate the performance of the proposed method when applied to the set of human proteins from the gold standard. We performed a 5-fold cross-validation to assess the combined and individual contributions of the clusters of features. Table 1 shows the results for the performance of each individual cluster while Table 2 presents the contribution of each cluster to the final classifier by iteratively removing each cluster.

**Table 1:** Analysis of the prediction performance of individual features

**Feature**	**AUC**	**CA**	**F1-score**	**Precision**	**Recall**
+ Literature	0.781	0.722	0.723	0.721	0.726
+ Sequence	0.877	0.784	0.790	0.768	0.813
+ GO	0.817	0.742	0.748	0.735	0.760
+ COGs	0.663	0.652	0.537	0.806	0.402
+ DDIs	0.620	0.617	0.424	0.861	0.281
Final Model	0.926	0.850	0.851	0.848	0.854

For each line the metrics are obtained by considering only that cluster of features on the classifier. *AUC*, area under the receiver operating characteristic (ROC) curve; *CA*, classification accuracy.

**Table 2:** Analysis of the contribution to the overall performance of individual cluster of features

**Feature**	**AUC**	**CA**	**F1-score**	**Precision**	**Recall**
- Literature	0.919	0.841	0.841	0.841	0.841
- Sequence	0.891	0.794	0.774	0.855	0.708
- GO	0.916	0.838	0.839	0.835	0.842
- COGs	0.923	0.846	0.847	0.842	0.852
- DDIs	0.911	0.831	0.834	0.819	0.850
Final Model	0.926	0.850	0.851	0.848	0.854

For each line the metrics are obtained by removing that cluster of features from the classifier. *AUC*, area under the receiver operating characteristic (ROC) curve; *CA*, classification accuracy.

The best performance is achieved through the ensemble of the five clusters, returning an area under the receiver operating characteristic (ROC) curve (AUC) of 0.926, a precision of 0.848 and a recall of 0.854. This result is above the performance of any individual feature and can only be achieved with the participation of all, meaning that all features are required and have a complementary contribution.

The Sequence is simultaneously the feature with the best overall performance (AUC = 0.877) and the one that causes the most negative impact when removed from the classifier, making the AUC drop to 0.891. It also has a very interesting recall of 0.813, partially due to the fact that all protein sequences are recognized and therefore the feature has full coverage.

In contrast, the clusters of orthologous groups (COGs, with AUC = 0.663) and domain-domain interactions (DDI, with AUC = 0.620) have the lowest individual AUCs, mainly due to the low coverage of their features. Despite that, they benefit from a considerably high precision that contributes positively to the final classifier. This is especially true for the COGs which, when removed, cause the major drop in precision.

The Literature and the Gene Ontology (GO) features, while not outstanding in any particular metric, have consistent performance on almost all metrics. Nevertheless, they make a very relevant contribution to the final classifier while the removal of the Literature causes a drop of the AUC to 0.919 and the GOs to 0.916.

### Global characterization of the human-microbial interactome

**Figure 2:**
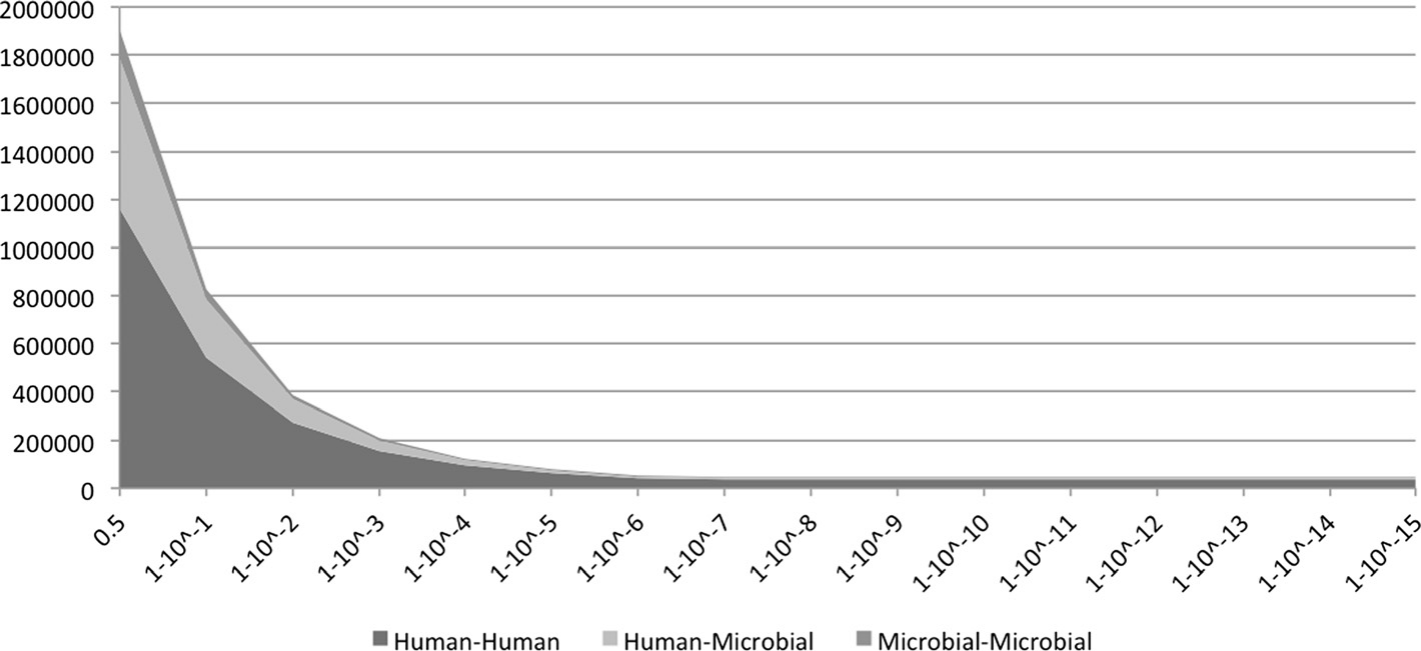
Plot with the relation of the number of interactions (y-axis) by classifier probability (x-axis).

**Figure 3:**
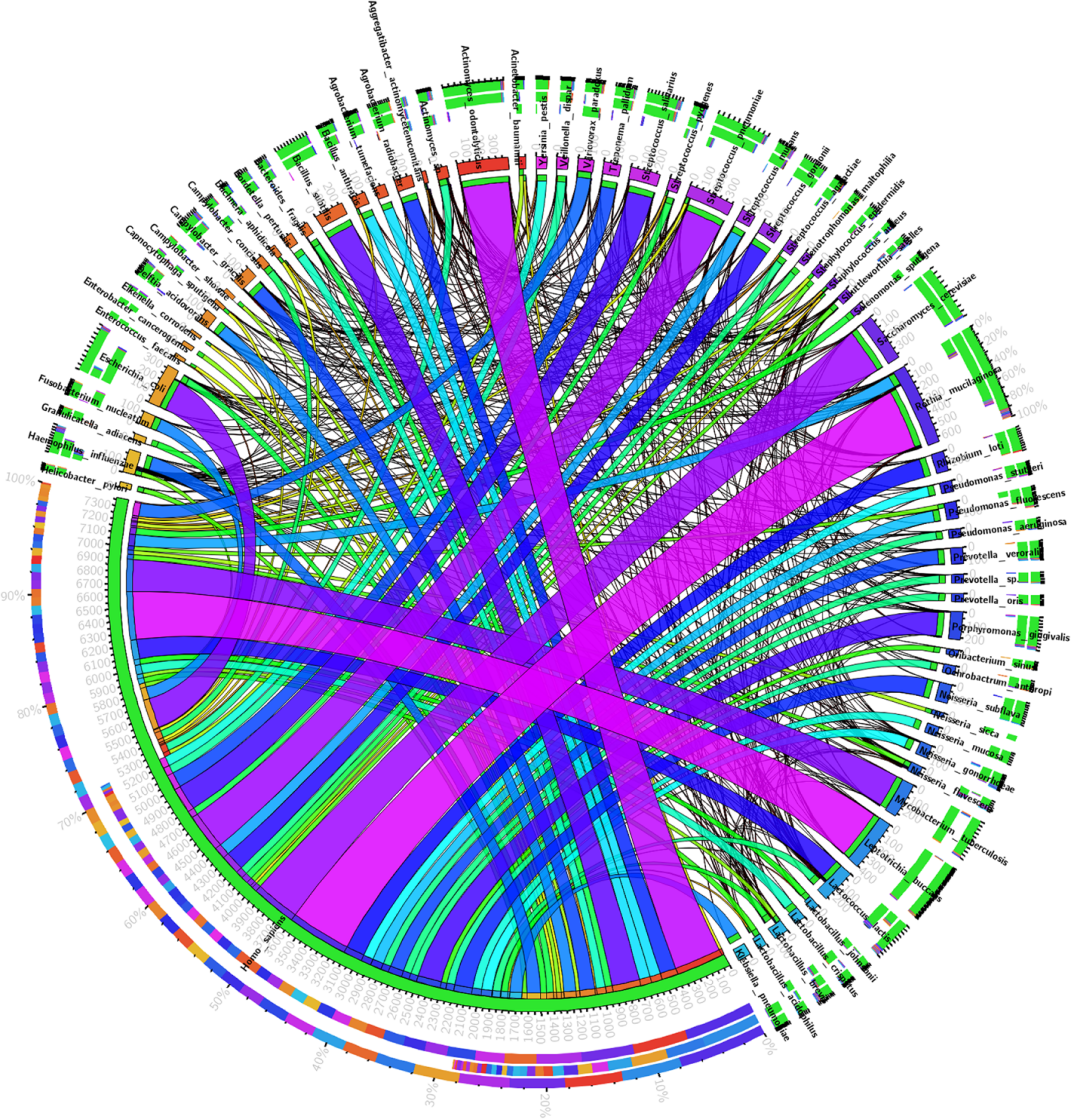
**Representation of the Human-microbial inter-species protein interactions.** Each section represents an organism. The ribbons connecting any two sections symbolize the PPIs between two organisms. The thickness of each ribbon correlates with the number of PPIs between both organisms.

The classifier returned a set of 1.9 million possible interactions with a probability higher than 0.5. This corresponds to an average degree of 404 interactions per protein, which is much above the range of 3 to 30 documented in previous studies [68]. Additionally, there are reports of yeast two-hybrid screenings, the most commonly used high-throughput experimental method, reaching false-positive rates of 70%. With this in mind, and in order to minimize the presence of false-positives in our predicted interactome, we filtered our prediction results to consider only very high confidence PPIs (probability ≥ 1-10^−7^). We neglected the recall for the sake of precision. As can be observed in Figure 2, the cutoff of 1-10^−7^ is the lowest probability value where an increment does not imply a decrease in the number of interactions. This cut-off resulted in a total of 46,579 PPIs, with 37,407 being between human proteins, 6,394 between human and microbial proteins, and 2,778 between microbial proteins. The average number of protein interactions per protein after the cutoff was 8. Figure 3 is a visual representation of the interactions between the various organisms found in the oral cavity and the human host. Intra-species interactions are not shown. The thickness of the ribbons between each organism is correlated with the number of PPIs between both organisms, meaning that the organisms sharing highest number of PPIs with the human are *Rothia mucilaginosa*, *Leptotrichia buccalis*, and *Actinomyces odontolyticus* (strain independent).

With the exception of *Homo sapiens* with 3,030 proteins, the most represented organisms in the human oral cavity are *Rothia mucilaginosa* (strain DY-18) (*Stomatococcus mucilaginosus*), *Actinomyces odontolyticus* (strain ATCC 17982), and *Streptococcus salivarius* (strain SK126), with 68, 54, and 26 proteins, respectively. These organisms are opportunistic pathogens known to be associated with periodontitis [69] and caries [70].

The most frequent biological processes are related to host-microbial interactions: GO:0044281 (small molecule metabolic process, involved in 173 PPIs), GO:0019048 (viral interaction with host, involved in 161 PPIs), and GO:0045087 (innate immune response, involved in 145 PPIs).

We also identified the top three human hub-proteins present in our data: epidermal growth factor receptor (EGFR) (UniProt AC P00533, involved in 3247 PPIs), fibronectin (UniProt AC P02751, involved in 3143 PPIs), and cullin-associated NEDD8-dissociated protein 1 (CAND1) (UniProt AC Q86VP6, involved in 2911 PPIs). In terms of non-human original hub-proteins, the most common are a serine/threonine protein kinase from *Leptotrichia buccalis* (UniProt AC C7NEK0, involved in 258 PPIs), a kinase domain protein from *Parviromonas micra* ATCC 33270 (UniProt AC A8SM03, involved in 194 PPIs), and Ras-related protein SEC4 from *Saccharomyces cerevisiae* (UniProt AC P07560, involved in 163 PPIs).

## Discussion

### Functional analysis of the human-microbial interactome

Unsurprisingly, the most frequent GO biological processes in our final PPI dataset are associated with host-pathogen interactions. The preeminence of innate immune response and viral interaction with host as the most frequent biological processes are self-explanatory. However, the association between small molecule metabolism and host-microbial interactions is not so direct.

When faced with an infection, the body will respond by initiating two major cellular signaling pathways with opposing functions: the nuclear factor (NF)-kB and glucocorticoid-mediated signal transduction cascades. While the NF-kB pathway promotes the immune response and inflammation, the glucocorticoid-mediated signal transduction cascade suppresses it. In order to explain the association between small molecule metabolism and host-pathogen interactions we must focus on the NF-kB cascade, as it is known to mediate the transcriptional activation of several cytokines (cell-signaling molecules) involved in immunity [71]. Tumor necrosis factor (TNF)-α and TNF-β, two of these cytokines, play key roles in immune regulation and inflammation [72]. However, these cytokines are mainly responsible for the metabolic instabilities that occur during the infection, as they increase the metabolism of triglycerides inducing hyperlipidemia (escalation of blood lipid levels), stimulate lipolysis (degradation of lipids), accelerate glycogen breakdown and glucose consumption and uptake, and increase the serum levels of hormones that regulate glucose metabolism. These metabolic changes possibly explain the great number of “small molecule metabolic process” biological processes.

### Analysis of hub proteins

The top three hub proteins identified share a common trait: these are exploited by pathogens in an attempt to gain entry to the host and survive inside it.

EGFR is a transmembrane protein mainly produced in the salivary glands and the kidneys [73]. Its association with microbial invasion has already been reported for *Salmonella typhimurium*[74], *Candida albicans*[75], *Reovirus*[76], and *Vaccinia virus*[77]. Apparently, all these pathogens initiate cellular invasion, at least to some extent, by binding to EGFR. This suggests the possibility that several other pathogens are using the EGFR to start host colonization, as supported by Buret *et al*. [78].

Similarly to EGFR, fibronectin appears to also play the role of a “microbial-anchor”. This glycoprotein is found bound to the β_1_ integrins in the cell surface, and is generally seen as a key protein for bacterial adhesion within the oral cavity [79,80].

The CAND1 protein, formerly TIP120A, was found to interact with most of the proteins in the Cullin family [81]. The Cullin protein family plays a key role in the ubiquitination of cellular proteins, i.e. performing a post-translational modification in order to label the target protein with ubiquitin molecules. This labeling frequently results in the commitment of the ubiquitin-linked protein to proteasomal degradation [82]. Consequently, CAND1 was suggested to function as a global regulator of cullin-containing ubiquitin ligases [81,83]. Being one of the top hub-proteins, we investigated the relationship between the ubiquitination pathway and pathogen colonization of the host cells. As expected, we found that certain bacteria corrupt the ubiquitination machinery as a means of regulating their virulence factors, or to trigger internalization of bacteria into host cells [84]. Such a mechanism improves the survival and replication chances of bacteria inside the host.

### Study of the microbiome role in periodontitis

When the data analysis is focused on a particular disease such as periodontal disease four main features can be observed: i) *Rothia mucilaginosa*, a microorganism present in the normal human oral microbiome but considered an opportunistic pathogen [85], is the species with the most interactions, with some of them revealing important and specific interactions; ii) new interactions are predicted between periodonto-pathogens and the host, and; iii) interactions between periodonto-pathogens are also predicted, most likely explaining a synergistic course of action, as has been previously proposed [86].

Regarding the first observation, the analysis of the sub-network pertaining to *Rothia mucilaginosa* shares the characteristics previously described for the hub proteins with 37/638 interactions with the EFGR protein, 40/638 interactions with fibronectin and 34/638 interactions with CAND1. Furthermore, this sub-network presents two predicted interactions which have not been described before: *R. mucilaginosa* proteins D2NSF5 and C6R5R8, which are predicted to interact with human immunoglobulin chains (P01719 and P01781), and could be related to the immune response specific for this species, explaining why these interactions are worth investigating.

If we consider the bacteria most associated with periodontal disease, our model predicts few interactions between *A. actinomycemcomitans*, *P. gingivalis*, and the host proteins. As mentioned before, this is due to the fact that these organisms are not well represented in the original protein data set. However, besides the interactions predicted between these bacteria and the human hub proteins described above, in the case of *Porphyromonas gingivalis* it is possible to identify at least two potentially interesting new types of interactions between bacterial ribosomal proteins and a major histocompatibility complex protein (P30461). Furthermore, we also identified a possible interaction between the bacterial enolase (Q7MTV8) and a host aquaporin which could interfere with the homeostasis mechanisms of the host. Additionally, when we consider the interactions of *P. gingivalis* with other bacteria, we find that the same enolase might interact with outer membrane proteins of *Haemophilus influenza* and *Pasteurella multocida*. The role of bacterial enolase as a multitask protein involved not only in carbohydrate metabolism but also in virulence has been recognized recently [87].

This suggests that previously unknown and important PPIs for oral colonization and biofilm formation may be present in this dataset. Finally the fact there are possible interactions between *P.gingivalis* proteases and those of other periodonto-pathogens such as *Kingella oralis* and *Treponema denticola* is interesting. This may even shed some light on the synergistic aspects of oral biofilm in periodontal disease [86].

## Conclusion

The continuous yield of large-scale data mainly from microarrays and yeast two-hybrid studies has made the study of PPIs very appealing. The main issue associated with PPI study is the high prevalence of false positives and negatives in experimental PPI data. Being the only “reliable” source of PPIs, inaccurate experimental PPI data will contaminate training datasets and therefore compromise the performance of computational PPI prediction methods. For this reason, we believe that an improvement in the quality of experimental PPI data will greatly impact the performance of new computational PPI prediction approaches. While this is not the case at present, we must consider how to avoid the effects of false positives and false negatives in the final PPI prediction model.

We proposed a probabilistic Bayesian-based method to integrate several data sources, to obtain more robust and reliable PPI predictions. By applying naïve Bayes, we automatically up-weigh the most informative features and down-weigh the less informative ones, allowing for automatic error-correction.

Our individual feature analysis results show a great relevance of the selected features. When applied on a naïve Bayes classifier, the individual features synergize, boosting the AUC up to 0.926. This suggests that the reliability of prediction improves with the increase of significant features, meaning that the ensemble final model actually reduces the disadvantages of the individual methods.

Cytoscape was successfully used to validate the network when tested with real pathway examples, discovering new potentially interesting interactions in oral biology, both between the host and the periodontal pathogens and between different periodontal pathogens.

We believe our work may be applied in several scientific areas, and even in other PPI related studies. An example is biomedical PPI screening, to assess if interactions of particular interest might occur and what the related interaction probability is. Another example is pharmacologic research, as a well-established PPI network can provide insights on potential drug targets, but also new uses for existent in-market drugs. Finally, and based on the fact that protein interaction networks are dynamic [88], our work can support researchers in identifying evolutionary patterns.

## Methods

### Oral proteome

As a starting point for our study we used 4,707 proteins, 3500 from Human and 1207 from microbial, available on the OralCard database [66,67].

These proteins were identified via proteomic analysis of the saliva, frequently by using 2D electrophoresis/mass spectrometry or 2D liquid chromatography/mass spectrometry. By the end of 2012 the salivary proteome was determined to contain 3500 proteins from human origin and 1207 from microbial sources.

### Predictive dataset construction

The use of positive (interacting pairs of proteins) and negative (non-interacting pairs of proteins) examples is required for training and assessing the performance of the classifier. All data used in the construction of the positive data set (PDS) and the negative data set (NDS) was downloaded in March 2013.

#### Positive dataset

**Figure 4:**
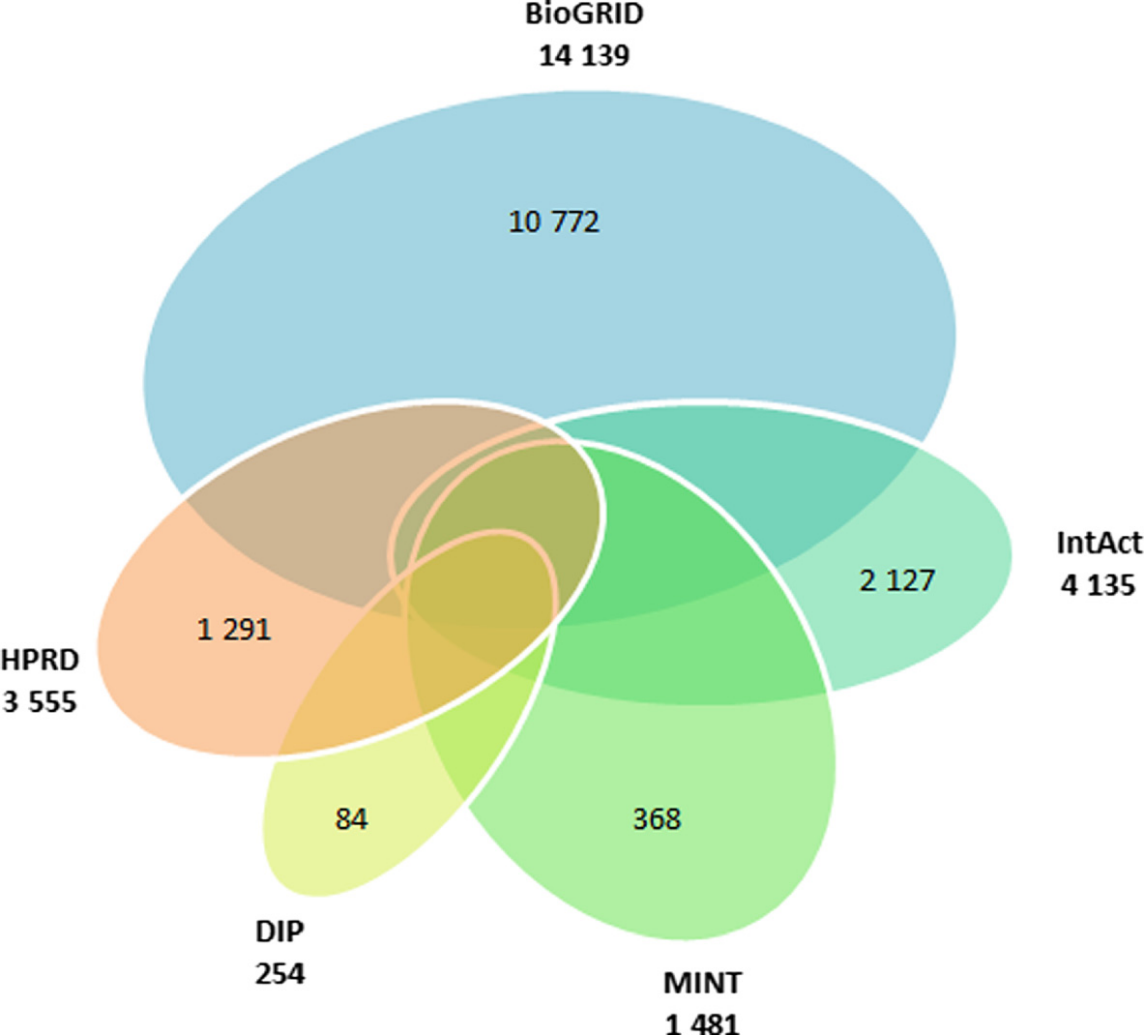
Venn diagram representing the intersections between the five high-quality experimentally determined protein-protein interaction databases.

We collected experimental oral protein-protein interaction (PPI) data from five databases: 14,139 PPIs from BIOGRID [89], 254 PPIs from DIP [90], 3,555 PPIs from HPRD [91], 4,135 PPIs from IntAct [92], and 1,481 PPIs from MINT [93], totaling 23,564 protein interactions (Figure 4).

All the interacting protein pairs were identified by their UniProtKB [94] Accession IDs for normalization purposes. In some instances it was necessary to convert the database own identifiers to UniProtKB Accession IDs. The BioGRID database represents interacting protein pairs using their own identifiers and Entrez Gene IDs. To match them to UniProtKB AccessionIDs we extracted the Gene IDs from the protein pairs and downloaded the list of respective gene products in the UniProtKB Accession ID format. UniProtKB allows direct mapping from the MINT and DIP databases to another identifier. A list of PPI pairs from both databases was uploaded to the UniProtKB mapping feature, resulting in two different sets of UniProtKB Accession ID pairs. HPRD uses its own identification system coupled with NCBI Reference Sequence Accession IDs (RefSeq) to classify PPI pairs. All the RefSeq Protein IDs were converted to UniProtKB Accession IDs and paired accordingly. IntAct PPI pairs are identified with UniProtKB Accession IDs and were directly extracted.

PPI pairs from the five databases were merged and repeated entries were removed. From a total of 23,564 PPIs, 5,193 duplicated entries were removed, resulting in a PDS of 18,371 protein pairs.

#### Negative dataset

The selection of negative examples to integrate the negative data was based on two methods described in the literature [95]. These methods consist of randomly selecting protein pairs that are not present in a veto list containing all PPIs from the positive data set. The use of this strategy was considered acceptable because the probability of committing an error while picking a random pair is low:

$$P\left(e\right)=\frac{N\times K}{N\times \left(N-1\right)}=\frac{K}{N-1},\left(K\ll N\right)\Rightarrow P\left(e\right)≅0,$$

where *N* is the number of proteins and *K* is the average degree for the final PPI network. In this study the *N* is 4,707 and for PPIs the typical value of *K* is between 6 and 16.

With this strategy we generated a NDS of a size similar to that of the PDS (18,348 “negative” protein pairs), and combined it with the PDS to obtain a training data set with 36,719 PPIs.

### Feature construction

In this section we describe the procedure for construction of the five clusters of features. The final results are summarized in Table 3.

**Table 3:** Relative coverage of protein-protein interactions present in the training and test data by individual feature clusters

	**Training data**		**Classification**	
	**#Interactions**	**% of total**	**#Interactions**	**% of total**
Literature	22,720	61.9%	4,698,390	69.9%
Sequence	35,379	96.4%	6,703,945	99.8%
GO	23,769	64.8%	5,130,103	76.4%
COGs	9,636	26.3%	1,324,230	19.7%
DDIs	5,994	16.3%	516,609	7.7%
Total	36,698	100.0%	6,716,792	100.0%

*GO*, gene ontology; *COGs*, clusters of orthologous groups; *DDIs*, domain-domain interactions.

#### Literature

The literature-based protein-protein interaction scores were calculated by the method described in van Haagen *et al.*[96]. This method is based on comparing the semantic contexts in which two proteins are mentioned in the published literature. The rationale for the method is that two proteins occurring in similar contexts will have a higher similarity score and are therefore more likely to interact. The semantic context for a given protein is defined by the concepts, from a pre-defined vocabulary, that are frequently mentioned in the same articles with that protein, and is represented by a vector containing a weight for each concept. These weights are based on the co-occurrence statistics, and measure the degree of association between the protein and each concept. Following Jelier *et al.*[97], we use the symmetric uncertainty coefficient *U* (*X*_*i*_*, Y*_*j*_) – where *X*_*i*_ is in this case the protein of interest and *Y*_*j*_ is any other concept in the vocabulary – as the weights used for creating the concept profiles:

$$U\left(X_{i},Y_{j}\right)=2\times \frac{H\left(Y_{j}\right)+H\left(X_{i}\right)-H\left(H_{i},Y_{j}\right)}{H\left(X_{i}\right)+H\left(Y_{j}\right)},$$

Where *H* (*X*) is the entropy for X and *H* (*X, Y*) is the joint entropy for *X* and *Y*, calculated based on document frequency counts.

We used a corpus of nearly one million abstracts, obtained by searching Pubmed with 17,402 names and synonyms extracted from UniProtKB for 4,707 proteins in the dataset, after removing nonsensical names such as “uncharacterized protein”. To identify the concepts mentioned in the texts we used Gimli [98], a machine-learning tool for gene and protein name recognition, together with dictionary matching to recognize other concepts from ten different semantic types including chemical entities, anatomical terms, diseases, pathways and GO terms. The dictionaries used contain around 1,3 million distinct names for around 400 thousand concepts. Based on the concept annotation of this corpus, we were able to calculate concept profiles for 22,720 protein pairs from the training dataset and 4,698,390 protein pairs for the classification dataset.

#### Primary protein sequence information

Several studies have been carried out where detection of protein-protein interaction is derived from information directly extracted from the amino-acid sequences [44-56]. The results indicate that the sequence information alone is sufficient to detect PPIs with reasonable accuracy [87] but may be improved if combined with other strategies.

Taking into account the primary protein sequence information, the following features have been considered in this work: occurrence of the 20 amino-acids in the protein sequence, protein atomic composition, molecular weight and atomic weight, forming a vector of 27 features. The interacting protein pair (*X, Y*) is represented by concatenating the corresponding features vectors *F*_*x*_ and *F*_*y*_, represented by (*F*_*x*_*, F*_*y*_).

We were able to obtain the sequence profile for 35,379 proteins pairs from the training dataset and 6,703,945 protein pairs for the classification dataset.

#### Orthologous profiles

By definition, clusters of orthologous groups (COGs) are sets of orthologous genes or orthologous groups of paralogs from three or more phylogenetic trees. In essence, this means that two proteins from different lineages belonging to the same COG are orthologous. Orthologs are genes in different species that evolved from a common ancestor by speciation (*i.e.* convergent evolution). In contrast, paralogs are genes related by duplication within a genome [99].

Lee *et al*. [100] aimed to expand the interactomes of various organisms by applying orthology-based methods in inter-species PPI prediction. They expanded orthologous pairs of 18 eukaryotic organisms and merged them with experimental PPI datasets, allowing the inference of PPIs for various species.

In this work we used the Search Tool for the Retrieval of Interacting Genes/Proteins (STRING) [101] database to obtain COGs and their respective combined scores. The combined score is computed by integrating the likelihoods from the different types of evidence, correcting for the probability of randomly observing an interaction [101]. This enhances the predictive performance of the method, as a combined score is only computed when more than one of the data sources in STRING supports a given association.

We were able to obtain the orthologous profile for 9,636 protein pairs from the training dataset and 1,324,230 proteins pairs for the classification dataset.

#### Biological process similarity

Previous studies have explored the use of GO annotation similarity between two proteins as a PPI predictor [59,102-105]. We downloaded biological process information from the GO Consortium [57] in March 2013 and calculated the depth of the GO terms (nodes) in the Directed Acyclic Graph (DAG), and the total number of proteins comprised between the smallest shared biological process (SSBP) for each pair of proteins and the following three branches. Since the depth of the GO terms in the DAG is implied in the total number of proteins, post-test odds analysis was performed solely on this feature to avoid redundancy. Such an approach was based on the general hypothesis that it is progressively more likely for the proteins comprised within a biological process to interact, if the total number of proteins involved in that process is progressively smaller.

We were able to obtain the gene ontology profile for 23,769 protein pairs from the training dataset and 5,130,103 protein pairs for the classification dataset.

#### Enriched conserved domain pairs

The Database of Protein Domain Interactions [106] (DOMINE) contains binary domain-domain interaction (DDI) data compiled from a collection of 15 databases and DDI prediction methods. Additionally, DOMINE provides a quality measure of the DDI confidence, as well as a binary classification of whether the domains are part of the same GO biological process. Here, we assume that whenever two given proteins possess one or more interacting domains between them, those proteins will interact. We adopted this DDI data collection as individual features in our approach. Since DOMINE provides DDI information from several sources, we tallied the number of sources that identified a DDI. This strategy confers higher reliability on DDI pairs with higher scores (closer to 15, the maximum number of DDI sources).

We were able to obtain the domain profile for 5,994 protein pairs from the training dataset and 516,609 protein pairs for the classification dataset.

### Data classification and validation

The proposed approach was developed, tested, optimized and performed using Orange, an open-source bioinformatics tool featuring Python scripting and a visual and programmatic interface. We used the naïve Bayes [107] classifier to predict PPIs in our data. The naïve Bayes classifier calculates the conditional probability of each attribute *A*_*i*_ given the class label *C*, from the training data. The Bayes rule is then applied to calculate the probability of *C* given the specific instance of *A*_*1*_ ,…, *A*_*n*_, and then assessing the class with the greatest posterior probability, ensuing classification [108].

The receiver operating characteristic (ROC) curve, which is the plot of the true positive (TP) rate with the false positive (FP) rate, depicting the relative trade-off between both rates [109] was used to evaluate the method’s performance. When comparing classifiers with very similar ROC curves, it may be necessary to estimate a single scalar value to represent the expected performance. One of the most common methods is calculation of the area under the ROC curve (AUC) [110], which we used to compare the naïve Bayes classifier. Therefore, we assessed the individual contributions of each feature in terms of classification accuracy (CA), area under curve (AUC), F1-score, precision and recall.

### Interactome analysis

We used Cytoscape to visualize and validate the obtained PPI network. The PPIs were classified as “HUMAN-HUMAN”, if the interacting proteins were only of human origin, as “MICRO-MICRO”, if the interacting proteins were only of microbial origin, or as “HUMAN-MICRO”.

We imported the network data to Cytoscape defining the two proteins in the same interacting protein pair as Source Interaction (protein one) and Target Interaction (protein two). The chosen Interaction Type was the above-mentioned organism-organism classification. A file containing node attributes was also imported, containing microorganism and biological process information extracted from the UniProt database pertaining to each individual protein in the network.

### Availability

All data required to analyze the results and re-run this experiment are available for download at http://bioinformatics.ua.pt/software/oralint. This includes the unique list of UniProt AC for the proteins in the oral cavity, the gold standard of interactions, the dataset used for training and validation, the predictions obtained, and the Cytoscape project file with the network.

## Competing interests

The authors declare that they have no competing interests.

## Authors’ contributions

EDC participated in the design of the study, constructed the positive and negative datasets, performed the analysis of hub proteins, characterized and analysed the human-microbial interactome, and drafted the manuscript. JPA conceived the study, participated in its design, performed feature construction and selection, parameterized the classifier, and helped to draft the manuscript. SM performed the text-mining analysis and helped to draft the manuscript. CP carried out primary protein sequence analysis and helped to draft the manuscript. NR and MJC analysed the role of the microbiome in periodontitis and helped to draft the manuscript. MB and JLO participated in the design and conception of the study, coordinated it, and helped to draft the manuscript. All authors read and approved the final manuscript.
